# Bound-state solutions and thermal properties of the modified Tietz–Hua potential

**DOI:** 10.1038/s41598-021-81428-9

**Published:** 2021-01-22

**Authors:** C. A. Onate, M. C. Onyeaju, E. Omugbe, I. B. Okon, O. E. Osafile

**Affiliations:** 1 Physics Programme, Department of Physical Sciences, Landmark University, Omu-Aran, Nigeria; 2 Theoretical Physics Group, Department of Physics, University of Port Harcourt, P.M.B. 5323, Choba, Port Harcourt, Nigeria; 3 Department of Physics, Federal University of Petroleum Resources, Effurun, Delta State Nigeria; 4 Theoretical Physics Group, Department of Physics, University of Uyo, Uyo, Nigeria

**Keywords:** Quantum physics, Statistical physics, thermodynamics and nonlinear dynamics, Physics

## Abstract

An approximate solutions of the radial Schrödinger equation was obtained under a modified Tietz–Hua potential via supersymmetric approach. The effect of the modified parameter and optimization parameter respectively on energy eigenvalues were graphically and numerically examined. The comparison of the energy eigenvalues of modified Tietz–Hua potential and the actual Tietz–Hua potential were examined. The ro-vibrational energy of four molecules were also presented numerically. The thermal properties of the modified Tietz–Hua potential were calculated and the effect of temperature on each of the thermal property were examined under hydrogen fluoride, hydrogen molecule and carbon (ii) oxide. The study reveals that for a very small value of the modified parameter, the energy eigenvalues of the modified Tietz–Hua potential and that of the actual Tietz–Hua potential are equivalent. Finally, the vibrational energies for Cesium molecule was calculated and compared with the observed value. The calculated results were found to be in good agreement with the observed value.

## Introduction

The study of any physical problem in quantum mechanics simply means finding the solution of second-order differential equation such as the Schrodinger wave equation that describes non-relativistic spinless particles and quantum–mechanical phenomena which dictates the dynamics of a quantum system. The solutions of this equation gives eigenvalues and eigenfunctions of the non-relativistic quantum system^1–4^. This solution is analytically exact and is limited to certain problems of spatial coordinate problems^5–7^. In most of the recent studies, the Schrödinger wave equation is used to describe the rotation-vibrational energies and the wave functions for diatomic molecules with diatomic molecule potential energy models. The expressions for the rotation-vibrational energy spectra and the wave functions for diatomic molecules play an important role in many areas, such as the calculations of rotational constants and centrifugal distortion constants^8^, computations of transition dipole matrix elements^9^, and calculations of thermal properties^10–12^, etc. The diatomic molecular potential energy function have applications in chemical physics and molecular physics as they provide the most compact way to summarize what is known about a molecule. Among the diatomic molecular potential energy function includes the generalized Morse potential, the Tietz potential, the Frost-Musulin potential, the Wei potential, the Morse potential, Rosen–Morse potential, the Tietz–Hua molecular potential and others. These potential models have received certain reports either in the relativistic regime or non-relativistic regime. The Tietz–Hua molecular potential function for instance, was studied by Falaye et al.^13^ under the non-relativistic and relativistic regime. These author calculated Fisher information entropy and some expectation values of the potential. The Tietz–Hua molecular potential function was also studied under Feinberg Horodecki equation by Ojonubah and Onate^14^. The explicit quantized momentum and the corresponding wave functions were deeply calculated. In one of the papers of Onate et al.^15^, the Tietz–Hua potential was modified and studied under the non-relativistic regime. The solutions of the non-relativistic equation was used to study Shannon entropy and information energy. The effect of the optimization parameter on energy and Shannon entropy were studied in detail. The authors failed to critically examined the relationship between the actual Tietz–Hua potential and the modified Tietz–Hua potential. They also failed to examine the effect of the modified parameter on energy. This gives an insight for another study on the potential.

As the interest in research studies increases day by day, researchers have decided to integrate similar areas of studies to foster wider applications of their research work. On this ground, statistical mechanics has been integrated into quantum mechanics via the thermodynamic properties. Several works on thermodynamic properties have been reported under different physical potential terms in the recent time. For instance, Oyewumi et al.^16^, studied the radial Schrӧdinger equation with Deng–Fan potential model, and also calculated the thermodynamic properties of the potential. The behaviour of the partition function, heat capacity, entropy, mean energy and free mean energy respectively against the temperature parameter were studied in detail for some diatomic molecules. Song et al.^17^ studied thermodynamic properties of sodium dimer under improved Rosen–Morse potential function through the computation of partition function. Their result is found to be in good agreement with the experimental value. Onate and Onyeaju^18^ in their own study, calculated thermodynamic properties of the Frost-Musulin potential via partition function. The exact and Poisson summation thermodynamic properties for diatomic molecules with Tietz potential was studied in ref.^19^. Recently the thermal functions for boron nitride with q-deformed exponential-type potential was obtained in ref.^20^. Dong and Cruz-Irisson^21^ in one of their works, obtained energy spectrum and thermodynamic properties of a modified Rosen–Morse potential model. The thermal properties were calculated via the vibrational partition function. This vibrational partition function also has applications in chemical physics and engineering e.g. modeling of the equilibrium constant of gas phase reaction^22^, examination of isotope fractionation during chemisorption reactions^23^ and calculation of the thermodynamic functions^24–26^. Motivated by the interest in thermodynamic properties and the modified Tietz–Hua potential, this study wants to examine the thermodynamic properties of the modified Tietz–Hua potential under some diatomic molecules using the methodology of supersymmetric approach.

**Figure 1:**
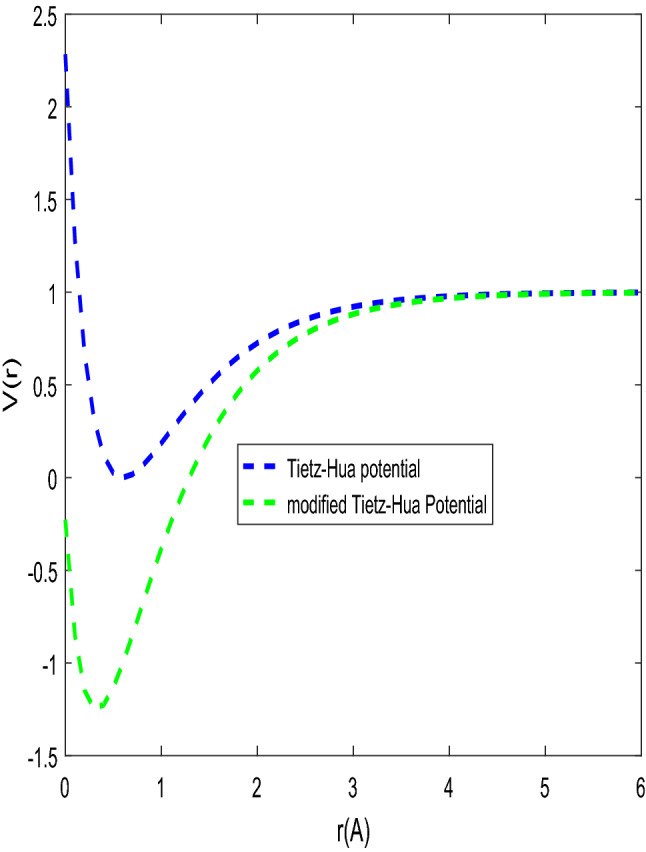
Tietz–Hua and modified Tietz–Hua potentials.

For quit some times, the ideas of SUSYQM have been applied to many quantum mechanical problems in both the relativistic and non-relativistic quantum mechanics. The path integral formulation of SUSYQM for instance was given in 1982 by Salomonson and van Holten^27^. After sometimes, some authors revealed that the tunneling rate through double-well barriers can be accurately solved via the methodology of SUSY^28–31^. With serious efforts, the ideas of SUSYQM has been introduced to systems of large numbers of particle and higher-dimensional systems. Recently, another useful concept known as shape-invariant potential was introduced by Gendenshtein^32^ who proved that the energy spectra for a shape-invariant potential can be deduced algebraically. Thus, the present work intends to investigate the thermal properties of some molecules under the modified Tietz–Hua potential via the susymmetric approach. The study will also extend to the computation of the vibrational energies of $Cs_{2} \left( {3^{3} \sum_{g}^{ + } } \right)$ molecule. The modified Tietz–Hua potential is given by^15^

1 $$ V(r) = D_{e} \left( {\frac{{C_{h} - 1}}{{1 - C_{h} e^{{ - b_{h} (r - r_{e} )}} }}} \right)be^{{ - b_{h} (r - r_{e} )}} + aD_{e} \left( {\frac{{1 - e^{{ - b_{h} (r - r_{e} )}} }}{{1 - C_{h} e^{{ - b_{h} (r - r_{e} )}} }}} \right)^{2} , $$

where $D_{e}$ is the dissociation energy, $r_{e}$ is equilibrium bond length, $C_{h}$ is optimization parameter, $a$ and $b$ are potential constants, $b_{h} = \beta (1 - C_{h} ),$ $\beta$ is Morse constant. The shape of the Tietz–Hua potential and modified Tietz–Hua potential are shown in Fig. 1.

The modified Tietz–Hua potential can be transform to other useful potentials by giving numerical value(s) to the potential parameters. When $b = 0,$ the modified part of Eq. (1) disappear completely leaving only the real Tietz–Hua potential as

2 $$ \mathop {\lim }\limits_{b \to 0} V(r) = aD_{e} \left( {\frac{{1 - e^{{ - b_{h} (r - r_{e} )}} }}{{1 - C_{h} e^{{ - b_{h} (r - r_{e} )}} }}} \right)^{2} . $$

When the potential parameter $a = 0,$ the actual Tietz–Hua potential goes off leaving the modified part of Eq. (1) as

3 $$ \mathop {\lim }\limits_{b \to 0} V(r) = D_{e} \left( {\frac{{C_{h} - 1}}{{1 - C_{h} e^{{ - b_{h} (r - r_{e} )}} }}} \right)be^{{ - b_{h} (r - r_{e} )}} . $$

When the optimization parameter $C_{h} = b = 0,$ the modified Tietz–Hua potential reduces to Morse potential of the form

4 $$ \mathop {\lim }\limits_{{C_{h} \to 0}} V(r) = D_{e} \left( {1 - e^{{ - b_{h} (r - r_{e} )}} } \right)^{2} . $$

The Morse potential accounts for the anharmonicity of real bonds and the non-zero transition probability for overtone and combination bands. It can also be used to model other interactions such as the interaction between an atom and a surface.

## Method

### Bound state solution

In this section, the solutions of the radial Schrӧdinger equation with the modified Tietz–Hua potential is obtained. To obtain the solutions for the modified Tietz–Hua potential, first, the radial Schrӧdinger equation with a centrifugal term is given as

5 $$ \left[ { - \frac{{\hbar^{2} }}{2\mu }\frac{{d^{2} }}{{dr^{2} }} - E_{n,\ell } + V(r) + \frac{{\hbar^{2} }}{2\mu }\frac{\ell (\ell + 1)}{{r^{2} }}} \right]U_{n,\ell } (r) = 0, $$

where $\hbar$ is the reduced Planck’s constant, $\mu$ is the reduced mass, $E_{n,\ell }$ is the non-relativistic energy, $V(r)$ is the interacting potential, $\ell$ is the angular momentum quantum number and $U_{n,\ell } (r)$ is the wave function. To obtain the solution of Eq. (5) for $\ell \ne 0,$ the centrifugal term must be approximated. Several approximation schemes have been adopted^33–35^ by different authors depending on the type of potential model under consideration. In this work, the centrifugal term will be approximated by the formula

6 $$ \frac{1}{{r^{2} }} \approx \frac{1}{{r_{e} }}\left( {D_{0} + \frac{{D_{1} }}{{e^{\alpha x} - C_{h} }} + \frac{{D_{1} }}{{(e^{\alpha x} - C_{h} )^{2} }}} \right), $$

where

7 $$ D_{0} = 1 + \frac{1}{\alpha }\left( {1 - C_{h} } \right)\left[ {3\left( {\frac{1}{\alpha } - 1} \right) - C_{h} \left( {\frac{3}{\alpha } + 1} \right)} \right], $$

8 $$ D_{1} = \frac{2}{\alpha }\left( {1 - C_{h} } \right)^{2} \left[ {2 + C_{h} + \frac{3}{\alpha }\left( {C_{h} - 1} \right)} \right], $$

9 $$ D_{2} = \frac{1}{\alpha }\left( {1 - C_{h} } \right)^{3} \left[ {\frac{3}{\alpha }\left( {1 - C_{h} } \right) - C_{h} - 1} \right]. $$

Substituting Eq. (1) and Eq. (6) into Eq. (5), the radial Schrӧdinger equation given in Eq. (5) turns to

10 $$ \frac{{d^{2} U_{n,\ell } (x)}}{{dx^{2} }} = \left[ {\frac{{V_{0} e^{ - \alpha x} }}{{1 - C_{h} e^{ - \alpha x} }} + \frac{{V_{1} - 2V_{1} e^{ - \alpha x} + V_{2} e^{ - 2\alpha x} }}{{(1 - C_{h} e^{ - \alpha x} )^{2} }} + \frac{{\ell (\ell + 1)D_{0} }}{{r_{e}^{2} }} - \frac{{2\mu E_{n,\ell } }}{{\hbar^{2} }}} \right]U_{n,\ell } (x), $$

where the following have been used for convenience

11 $$ V_{0} = \frac{{2\mu D_{e} b(C_{h} - 1)}}{{\hbar^{2} }} + \frac{{\ell (\ell + 1)D_{1} }}{{r_{e}^{2} }}, $$

12 $$ V_{1} = \frac{{2\mu aD_{e} }}{{\hbar^{2} }}, $$

13 $$ V_{2} = \frac{{2\mu aD_{e} }}{{\hbar^{2} }} + \frac{{\ell (\ell + 1)D_{2} }}{{r_{e}^{2} }}, $$

14 $$ \begin{aligned} \alpha & = b_{h} r_{e} , \\ x & = \frac{{r - r_{e} }}{{r_{e} }}. \\ \end{aligned} $$

At this point, the basic concepts of the supersymmetric quantum mechanics formalism^36^ and shape-invariance technique is applied to solve Eq. (10). In other to achieve this, the first step is to write the ground-state wave function of the form

15 $$ U_{0,\ell } (x) = exp\left( { - \int {W(x)dx} } \right), $$

where $W(x)$ is known as the superpotential function in supersymmetric quantum mechanics. The superpotential $W(x)$ is the solution of a Riccati equation that will soon be written. Substituting Eq. (15) into Eq. (10), we have a Riccati equation of the form

16 $$ W^{2} (x) - \frac{dW(x)}{{dx}} = \frac{{V_{0} e^{ - \alpha x} }}{{1 - C_{h} e^{ - \alpha x} }} + \frac{{V_{1} - 2V_{1} e^{ - \alpha x} + V_{2} e^{ - 2\alpha x} }}{{(1 - C_{h} e^{ - \alpha x} )^{2} }} + \frac{{\ell (\ell + 1)D_{0} }}{{r_{e}^{2} }} - \frac{{2\mu E_{n,\ell } }}{{\hbar^{2} }}. $$

In order to make the left hand side of Eq. (16) compatible with the property of the right hand side, a superpotential function is proposed as

17 $$ W(x) = \rho_{0} + \frac{{\rho_{1} e^{ - \alpha x} }}{{1 - e^{ - \alpha x} }}, $$

where $\rho_{0}$ and $\rho_{1}$ are superpotential parameters whose values will soon be determined. The present work will study the bound state solution whose radial part of the wave function $U_{n,\ell } (x)$ satisfy the boundary conditions that $U_{n,\ell } (x)/x$ becomes zero as $x \to \infty ,$ and $U_{n,\ell } (x)/x$ is finite at $x = 0.$ It is only when $x \to \infty ,$ $U_{n,\ell } (x)/x$ is finite and $U_{n,\ell } (x)/x = 0$ at the origin point $x = 0,$ the radial wave function can satisfy the boundary conditions. Substituting Eq. (17) into Eq. (16) with some mathematical manipulations, the values of the two superpotential parameters are obtain as

18 $$ \rho_{0}^{2} = \ell (\ell + 1)D_{0} + \frac{{2\mu (aD_{e} - E_{n,\ell } )}}{{\hbar^{2} }}, $$

19 $$ \rho_{1} = \frac{{r_{e}^{2} b_{h}^{2} C_{h} }}{2}\left( { - 1 \pm \sqrt {1 + \frac{{4\ell (\ell + 1)D_{2} }}{{r_{e}^{2} b_{h}^{2} C_{h}^{2} }} + \frac{{8\mu aD_{e} (1 - C_{h} )^{2} }}{{b_{h}^{2} C_{h}^{2} \hbar^{2} }}} } \right), $$

20 $$ \rho_{0} = \frac{{\frac{{2\mu D_{e} \left[ {b\left( {C_{h} - 1} \right) + a\left( {C_{h} - \frac{1}{{C_{h} }}} \right)} \right]}}{{\hbar^{2} b_{h}^{2} }} - \frac{\ell (\ell + 1)}{{C_{h}^{2} }}\left( {D_{2} - D_{1} C_{h} } \right) + \frac{{\rho_{1}^{2} }}{{C_{h} }}}}{{2\rho_{1} }}. $$

In terms of the superpotential function $W(x)$ in Eq. (17), the two partner potentials $V_{ \pm } (x) = W^{2} (x) \pm \frac{dW(x)}{{dx}}$ of the supersymmetric quantum mechanics can easily be written as

21 $$ V_{ + } (x) = \rho_{0}^{2} + \frac{{(2\rho_{0} \rho_{1} - \alpha \rho_{1} )e^{ - \alpha x} }}{{1 - C_{h} e^{ - \alpha x} }} + \frac{{B(B + \alpha C_{h} )e^{ - 2\alpha x} }}{{\left( {1 - C_{h} e^{ - \alpha x} } \right)^{2} }}, $$

22 $$ V_{ - } (x) = \rho_{0}^{2} + \frac{{(2\rho_{0} \rho_{1} + \alpha \rho_{1} )e^{ - \alpha x} }}{{1 - C_{h} e^{ - \alpha x} }} + \frac{{B(B + \alpha C_{h} )e^{ - 2\alpha x} }}{{\left( {1 - C_{h} e^{ - \alpha x} } \right)^{2} }}. $$

Putting $\rho_{1} = a_{0} ,$ it can easily be shown^37^ that the two partner potentials $V_{ + } (x)$ and $V_{ - } (x)$ are satisfied the following relationship

23 $$ V_{ + } (x,a_{0} ) = V_{ - } (x,a_{1} ) + R(a_{1} ), $$

where $a_{1}$ is a function of $a_{0} ,$ i.e. $a_{1} = a_{0} + \alpha ,$ and the residual term $R(a_{1} )$ is independent of the variable $x.$ In terms of the parameters of the system, the residual term can be express as

24 $$ R(a_{1} ) = \left( {\frac{{\rho_{R} + \frac{\ell (\ell + 1)}{{C_{h} }}\left( {D_{1} - \frac{{D_{2} }}{{C_{h} }}} \right) + \frac{{a_{0}^{2} }}{{C_{h} }}}}{{2a_{0} }}} \right)^{2} - \left( {\frac{{\rho_{R} + \frac{\ell (\ell + 1)}{{C_{h} }}\left( {D_{1} - \frac{{D_{2} }}{{C_{h} }}} \right) + \frac{{a_{1}^{2} }}{{C_{h} }}}}{{2a_{1} }}} \right)^{2} , $$

25 $$ R(a_{2} ) = \left( {\frac{{\rho_{R} + \frac{\ell (\ell + 1)}{{C_{h} }}\left( {D_{1} - \frac{{D_{2} }}{{C_{h} }}} \right) + \frac{{a_{1}^{2} }}{{C_{h} }}}}{{2a_{1} }}} \right)^{2} - \left( {\frac{{\rho_{R} + \frac{\ell (\ell + 1)}{{C_{h} }}\left( {D_{1} - \frac{{D_{2} }}{{C_{h} }}} \right) + \frac{{a_{2}^{2} }}{{C_{h} }}}}{{2a_{2} }}} \right)^{2} , $$

26 $$ R(a_{3} ) = \left( {\frac{{\rho_{R} + \frac{\ell (\ell + 1)}{{C_{h} }}\left( {D_{1} - \frac{{D_{2} }}{{C_{h} }}} \right) + \frac{{a_{2}^{2} }}{{C_{h} }}}}{{2a_{2} }}} \right)^{2} - \left( {\frac{{\rho_{R} + \frac{\ell (\ell + 1)}{{C_{h} }}\left( {D_{1} - \frac{{D_{2} }}{{C_{h} }}} \right) + \frac{{a_{3}^{2} }}{{C_{h} }}}}{{2a_{3} }}} \right)^{2} , $$

27 $$ R(a_{n} ) = \left( {\frac{{\rho_{R} + \frac{\ell (\ell + 1)}{{C_{h} }}\left( {D_{1} - \frac{{D_{2} }}{{C_{h} }}} \right) + \frac{{a_{n - 1}^{2} }}{{C_{h} }}}}{{2a_{n - 1} }}} \right)^{2} - \left( {\frac{{\rho_{R} + \frac{\ell (\ell + 1)}{{C_{h} }}\left( {D_{1} - \frac{{D_{2} }}{{C_{h} }}} \right) + \frac{{a_{n}^{2} }}{{C_{h} }}}}{{2a_{n} }}} \right)^{2} . $$

Following the shape-invariance approach, the energy eigenvalue can be determined via the consideration of the negative partner potential. Hence

28 $$ E_{0}^{( - )} = 0, $$

29 $$ E_{n}^{( - )} = \sum\limits_{\kappa = 1}^{n} {R(a_{\kappa } ) = } R(a_{1} ) + R(a_{2} ) + R(a_{3} ) + - - - + R(a_{n} ), $$

30 $$ \overline{E} = E_{0}^{( - )} + E_{n}^{( - )} = \left( {\frac{{\rho_{T} + \ell (\ell + 1)\left( {D_{1} - \frac{{D_{2} }}{{C_{h} }}} \right) + \frac{{a_{n}^{2} }}{{C_{h} }}}}{{2a_{n} }}} \right)^{2} . $$

This gives full energy eigenvalue equation as

31 $$ E_{n,\ell } = aD_{e} + \frac{{\hbar^{2} \ell (\ell + 1)D_{0} }}{{2\mu r_{e}^{2} }} - \frac{{b_{h}^{2} \hbar^{2} }}{2\mu }\left[ {\frac{{\rho_{R} - \frac{\ell (\ell + 1)}{{r_{e}^{2} b_{h}^{2} C_{h}^{2} }}\left( {D_{2} - D_{1} C_{h} } \right) + \left( {n + \frac{1}{2} + \frac{1}{2}\rho_{T} } \right)^{2} }}{{2\left( {n + \frac{1}{2} + \frac{1}{2}\rho_{T} } \right)}}} \right]^{2} . $$

32 $$ \rho_{R} = \frac{{2\mu D_{e} \left[ {b\left( {1 - \frac{1}{{C_{h} }}} \right) + a\left( {1 - \frac{1}{{C_{h}^{2} }}} \right)} \right]}}{{b_{h}^{2} \hbar^{2} }}, $$

33 $$ \rho_{T} = \sqrt {1 + \frac{{4\ell (\ell + 1)D_{2} }}{{r_{e}^{2} b_{h}^{2} C_{h}^{2} }} + \frac{{8\mu aD_{e} (1 - C_{h} )^{2} }}{{\hbar^{2} b_{h}^{2} C_{h}^{2} }}} . $$

Defining $y = e^{{ - b_{h} r_{e} \left( {\tfrac{{r - r_{e} }}{{r_{e} }}} \right)}} ,$ the radial wave function is obtain as

34 $$ U_{n,\ell } (y) = N_{n,\ell } y^{{\sqrt {\rho_{0}^{2} } }} (1 - C_{h} y)^{{\tfrac{1}{2} + \tfrac{1}{2}\sqrt {\rho_{T} } }} P_{n}^{{\left( {2\sqrt {\rho_{0}^{2} } ,\sqrt {\rho_{T} } } \right)}} \left( {1 - 2C_{h} y} \right). $$

### Thermal properties of modified Tietz–Hua potential

To calculate the thermodynamic properties, the energy eigenvalue equation in Eq. (31) is modify so that the vibrational energy becomes

35 $$ E_{n,\ell } = Q_{0} - Q_{1} \left( {n + \delta + \frac{{Q_{2} }}{n + \delta }} \right)^{2} , $$

where

36 $$ \begin{aligned} Q_{0} & = aD_{e} + \frac{{\hbar^{2} l\left( {l + 1} \right)D_{0} }}{{2\mu r_{e}^{2} }},Q_{1} = \frac{{b_{h}^{2} \hbar^{2} }}{8\mu } \\ \delta & = \frac{1}{2} + \frac{1}{2}\sqrt {1 + \frac{{4\ell (\ell + 1)D_{2} }}{{r_{e}^{2} b_{h}^{2} C_{h}^{2} }} + \frac{{8\mu aD_{e} (1 - C_{h} )^{2} }}{{\hbar^{2} b_{h}^{2} C_{h}^{2} }}} \\ Q_{2} & = \frac{{2\mu D_{e} \left[ {b(C_{h} - 1) + a\left( {1 - \frac{1}{{C_{h}^{2} }}} \right)} \right]}}{{b_{h}^{2} \hbar^{2} }} - \frac{\ell (\ell + 1)}{{r_{e}^{2} b_{h}^{2} C_{h}^{2} }}\left( {D_{2} - D_{1} C_{h} } \right). \\ \end{aligned} $$

If we let $\rho = n + \delta$ then (1) can be simplified further as

37 $$ E_{nl} = - \Lambda_{0} - \left( {Q_{1} \rho^{2} + \frac{{Q_{1} Q_{2}^{2} }}{{\rho^{2} }}} \right), $$

38 $$ \Lambda_{0} = 2Q_{1} Q_{2} - Q_{0} . $$

From (35), the maximum vibrational quantum number is obtained from $\frac{{dE_{n,0} }}{dn} = 0$, $v_{\max } = - \delta + \sqrt {Q_{2} }$.

Using Eq. (36), the partition function can be written as

39 $$ Z\left( \beta \right) = e^{{\beta \Lambda_{0} }} \int\limits_{\delta }^{{v_{\max } + \delta }} {e^{{\beta \left( {Q_{1} \rho^{2} + \frac{{Q_{1} Q_{2}^{2} }}{{\rho^{2} }}} \right)}} } d\rho = \frac{{{\text{e}}^{{ - \beta Q_{0} }} \sqrt {\uppi } \left[ {e^{{4\beta {\Lambda }_{1} }} \left[ {{\text{erf}}\left( {{\Lambda }_{2} \sqrt \beta } \right) - 1} \right] + \left[ {{\text{erf}}\left( {{\Lambda }_{3} \sqrt \beta } \right) + 1} \right]} \right]}}{{{\Lambda }_{4} \sqrt \beta }} $$

40 $$ {\Lambda }_{1} = {\text{Q}}_{1} {\text{Q}}_{2} , $$

41 $$ {\Lambda }_{2} = \sqrt { - {\text{Q}}_{1} } \left( {p + \frac{{{\text{Q}}_{2} }}{p}} \right),\quad \delta \le p \le v_{\max } + \delta , $$

42 $$ {\Lambda }_{3} = \sqrt { - {\text{Q}}_{1} } \left( {p + \frac{{{\text{Q}}_{2} }}{p}} \right),\quad \delta \le p \le v_{\max } + \delta , $$

43 $$ {\Lambda }_{4} = \sqrt { - 2Q_{3} } , $$

44 $$ Q_{3} = \frac{{b_{h}^{2} \hbar^{2} }}{\mu }, $$

- Vibration mean energy:
  45 $$ \begin{aligned} U\left( \beta \right) & = - \frac{\partial \ln Z\left( \beta \right)}{{\partial \beta }} = \frac{{{\Lambda }_{4} \sqrt \beta }}{{{\Lambda }_{4} \sqrt \beta \left[ {e^{{ - \beta Q_{0} }} \sqrt \pi e^{{4\beta \Delta_{1} }} \left( {erf\left( {{\Lambda }_{2} \sqrt \pi } \right) - 1} \right) + \left( {erf\left( {{\Lambda }_{3} \sqrt \beta } \right) + 1} \right)} \right]}} \\ & \quad \times \left[ \begin{gathered} \frac{{(1 + Q_{0} )\left( {e^{{ - \beta Q_{0} }} \sqrt \pi e^{{4\beta {\Lambda }_{1} }} \left( {erf\left( {{\Lambda }_{2} \sqrt \pi } \right) - 1} \right) + \left( {erf\left( {{\Lambda }_{3} \sqrt \beta } \right) + 1} \right)} \right)}}{{{\Lambda }_{4} \sqrt \beta }} + \frac{{\frac{{e^{{ - \beta {\Lambda }_{3}^{2} }} {\Lambda }_{3} }}{\sqrt \pi \sqrt \beta }}}{{{\Lambda }_{4} \sqrt \beta }} \hfill \\ - \frac{{e^{{ - \beta Q_{0} }} \sqrt \pi e^{{4\beta {\Lambda }_{1} }} \left( {erf\left( {{\Lambda }_{2} \sqrt \pi } \right) - 1} \right) + \left( {erf\left( {{\Lambda }_{3} \sqrt \beta } \right) + 1} \right)}}{{2{\Lambda }_{4} \beta^{3/2} }} + \frac{{\frac{{e^{{\beta {\Lambda }_{1} \left( {4 + {\Lambda }_{1} } \right)}} {\Lambda }_{2} }}{\sqrt \pi \sqrt \beta }}}{{{\Lambda }_{4} \sqrt \beta }} \hfill \\ \end{gathered} \right]. \\ \end{aligned} $$
- Vibrational specific heat capacity:
  46 $$ \begin{aligned} C\left( \beta \right) & = k\beta^{2} \left( { \frac{{\partial^{2} \ln Z\left( \beta \right)}}{{\partial \beta^{2} }}} \right) \\ & = Q_{0} \left( {e^{{ - \beta Q_{0} }} \sqrt \pi e^{{4\beta {\Lambda }_{1} }} \left( {erf\left( {{\Lambda }_{2} \sqrt \pi } \right) - 1} \right) + \left( {erf\left( {{\Lambda }_{3} \sqrt \beta } \right) + 1} \right)} \right)\left( {A_{1} Q_{0} - A_{2} Q_{0} + A_{3} } \right) - A_{1} \frac{{e^{{\beta \left( {4{\Lambda }_{1} - {\Lambda }_{2}^{2} } \right)}} {\Lambda }_{2} }}{{2\sqrt \pi \beta^{3/2} }} \\ & \quad - 2A_{1} Q_{0} e^{{ - \beta Q_{0} }} \sqrt \pi e^{{4\beta {\Lambda }_{1} }} \left( {erf\left( {{\Lambda }_{2} \sqrt \pi } \right) - 1} \right) + \frac{{e^{{\beta {\Lambda }_{1} \left( {4 + {\Lambda }_{1} } \right)}} {\Lambda }_{2} + e^{{ - \beta {\Lambda }_{3}^{2} }} {\Lambda }_{3} }}{\sqrt \pi \sqrt \beta } - A_{1} \frac{{e^{{ - \beta {\Lambda }_{3}^{2} (1 + {\Lambda }_{3} )}} {\Lambda }_{3} {(1} + 2\beta^{ - 1} {\Lambda }^{2} )}}{{2\sqrt \pi \beta^{3/2} }} \\ & \quad + \frac{{e^{{ - \beta Q_{0} }} \sqrt \pi e^{{4\beta {\Lambda }_{1} }} \left( {erf\left( {{\Lambda }_{2} \sqrt \pi } \right) - 1} \right) + \left( {erf\left( {{\Lambda }_{3} \sqrt \beta } \right) + 1} \right)}}{\beta }\left( {A_{1} - A_{4} \left( {1 + \frac{1}{2\sqrt \beta }} \right) + \frac{{A_{3} - A_{2} Q_{0} }}{2}} \right) \\ & \quad + A_{1} e^{{ - \beta Q_{0} }} \sqrt \pi \left( {{4}\Lambda_{1} e^{{4\beta {\Lambda }_{1} }} \left( {erf\left( {{\Lambda }_{2} \sqrt \pi } \right) - 1} \right)} \right)\left( {4{\Lambda }_{1} - \frac{1}{\beta }} \right) + A_{1} \frac{{8{\Lambda }_{1} e^{{\beta \left( {4{\Lambda }_{1} - {\Lambda }_{2}^{2} } \right)}} {\Lambda }_{2} - e^{{\beta \left( {4{\Lambda }_{1} - {\Lambda }_{2}^{2} } \right)}} {\Lambda }_{2}^{3} }}{\sqrt \pi \sqrt \beta } \\ & \quad + A_{1} \beta^{2} \frac{{3e^{{ - \beta Q_{0} }} \sqrt \pi \left( {e^{{4\beta {\Lambda }_{1} }} \left( {erf\left( {{\Lambda }_{2} \sqrt \pi } \right) - 1} \right) + erf\left( {{\Lambda }_{3} \sqrt \beta } \right) - 1} \right)}}{4} + A_{1} \frac{{\frac{{{\Lambda }_{2} e^{{\beta \left( {4{\Lambda }_{1} - {\Lambda }_{2}^{2} } \right)}} + e^{{ - \beta {\Lambda }_{3}^{2} }} {\Lambda }_{3} }}{\sqrt \pi \sqrt \beta }}}{\beta } \\ & \quad + \frac{{e^{{ - \beta Q_{0} }} \sqrt \pi \left( {4{\Lambda }_{1} e^{{4\beta {\Lambda }_{1} }} \left( {erf\left( {{\Lambda }_{2} \sqrt \pi } \right) - 1} \right)} \right) + \frac{{e^{{\beta \left( {4\Lambda_{1} - \Lambda_{2}^{2} } \right)}} {\Lambda }_{2} + e^{{ - \beta {\Lambda }_{3}^{2} }} {\Lambda }_{3} }}{\sqrt \pi \sqrt \beta }}}{\beta }\left( {A_{2} Q_{0} - A_{3} + A_{4} } \right) \\ \end{aligned} $$
- Vibrational free energy:
  47 $$ F\left( \beta \right) = - {\text{kT ln}}Z\left( \beta \right) = \frac{{ - \ln \left[ {\frac{{e^{{ - \beta Q_{0} }} \sqrt \pi e^{{4\beta {\Lambda }_{1} }} \left( {erf\left( {{\Lambda }_{2} \sqrt \pi } \right) - 1} \right) + \left( {erf\left( {{\Lambda }_{3} \sqrt \beta } \right) + 1} \right)}}{{{\Lambda }_{4} \sqrt \beta }}} \right]}}{\beta }. $$
- Vibrational entropy:
  48 $$ \begin{aligned} S\left( \beta \right) & = {\text{k ln}}Z\left( \beta \right) - k\beta \frac{\partial \ln Z\left( \beta \right)}{{\partial \beta }} \\ & = \left[ \begin{gathered} - A_{4} \frac{{e^{{ - \beta Q_{0} }} \sqrt \pi \left( {{4}\Lambda_{1} e^{{4\beta {\Lambda }_{1} }} \left( {erf\left( {{\Lambda }_{2} \sqrt \pi } \right) - 1} \right)} \right) + \left( {erf\left( {{\Lambda }_{3} \sqrt \pi } \right) + 1} \right)}}{{2{\Lambda }_{4} \sqrt \beta }}\left( {1 + \frac{1}{2\beta }} \right) \hfill \\ + A_{4} \frac{{{\Lambda }_{4} e^{{ - \beta Q_{0} }} \sqrt \pi \left( {{4}\Lambda_{1} e^{{4\beta {\Lambda }_{1} }} \left( {erf\left( {{\Lambda }_{2} \sqrt \pi } \right) - 1} \right)} \right) + \frac{{8{\Lambda }_{4} {\Lambda }_{1} e^{{\beta \left( {4{\Lambda }_{1} - {\Lambda }_{2}^{2} } \right)}} {\Lambda }_{2} + e^{{ - \beta {\Lambda }_{3}^{2} }} {\Lambda }_{3} }}{\sqrt \pi \sqrt \beta }}}{{{2}\beta^{3/2} }} \hfill \\ \end{gathered} \right]. \\ \end{aligned} $$
  49 $$ A_{1} = \frac{{{\Lambda }_{4} \sqrt \beta }}{{e^{{ - \beta Q_{0} }} \sqrt \pi \left( {{4}\Lambda_{1} e^{{4\beta {\Lambda }_{1} }} \left( {erf\left( {{\Lambda }_{2} \sqrt \pi } \right) - 1} \right)} \right) + \left( {erf\left( {{\Lambda }_{3} \sqrt \pi } \right) + 1} \right)}}, $$
  50 $$ A_{2} = \frac{1}{{e^{{ - \beta Q_{0} }} \sqrt \pi \left( {{4}\Lambda_{1} e^{{4\beta {\Lambda }_{1} }} \left( {erf\left( {{\Lambda }_{2} \sqrt \pi } \right) - 1} \right)} \right) + \left( {erf\left( {{\Lambda }_{3} \sqrt \pi } \right) + 1} \right)}}, $$
  51 $$ A_{3} = \left[ {\frac{{{\Lambda }_{4} e^{{ - \beta Q_{0} }} \sqrt \pi \left( {{4}\Lambda_{1} e^{{4\beta {\Lambda }_{1} }} \left( {erf\left( {{\Lambda }_{2} \sqrt \pi } \right) - 1} \right)} \right) + \frac{{8{\Lambda }_{4} {\Lambda }_{1} e^{{\beta \left( {4{\Lambda }_{1} - {\Lambda }_{2}^{2} } \right)}} {\Lambda }_{2} + e^{{ - \beta {\Lambda }_{3}^{2} }} {\Lambda }_{3} }}{\sqrt \pi \sqrt \beta }}}{{e^{{ - \beta Q_{0} }} \beta \sqrt \pi \left( {{4}\Lambda_{1} e^{{4\beta {\Lambda }_{1} }} \left( {erf\left( {{\Lambda }_{2} \sqrt \pi } \right) - 1} \right)} \right) + \left( {erf\left( {{\Lambda }_{3} \sqrt \pi } \right) + 1} \right)\beta }}} \right]^{2} . $$
  52 $$ A_{4} = \frac{{{\Lambda }_{4} }}{{e^{{ - \beta Q_{0} }} \sqrt \pi \left( {{4}\Lambda_{1} e^{{4\beta {\Lambda }_{1} }} \left( {erf\left( {{\Lambda }_{2} \sqrt \pi } \right) - 1} \right)} \right) + \left( {erf\left( {{\Lambda }_{3} \sqrt \pi } \right) + 1} \right)}}. $$

## Discussion of result

**Figure 2:**
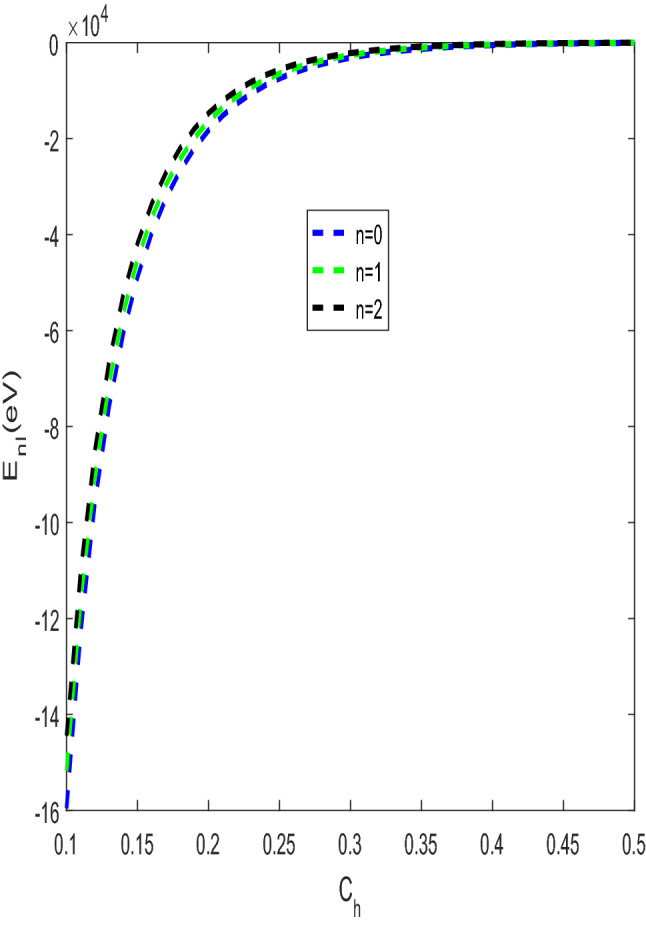
Energy $E_{n,\ell }$ against the $C_{h}$ with $\mu = \hbar = \ell = 1,$ $b_{h} = 0.5$ Å, $r_{e} = 0.5$ Å, $b = 0.001$ Å and $D_{e} = 10\;{\text{eV}}$.

**Figure 3:**
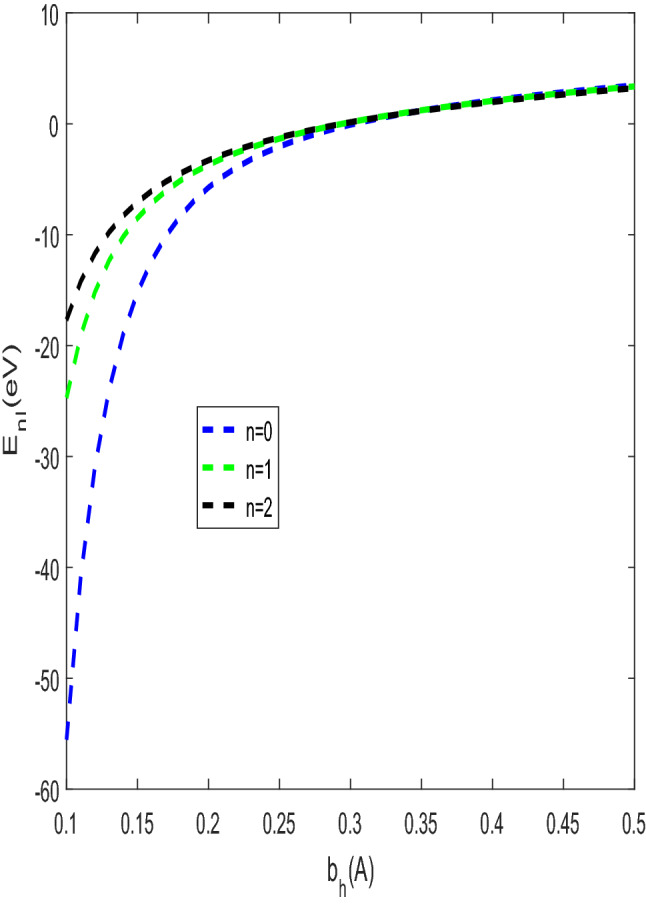
Energy $E_{n,\ell }$ against the $b_{h}$ with $\mu = \hbar = \ell = 1,$ $b = 0.001$ Å, $C_{h} = 0.95,$ $r_{e} = 0.5$ Å and $D_{e} = 10\;{\text{eV}}.$

**Figure 4:**
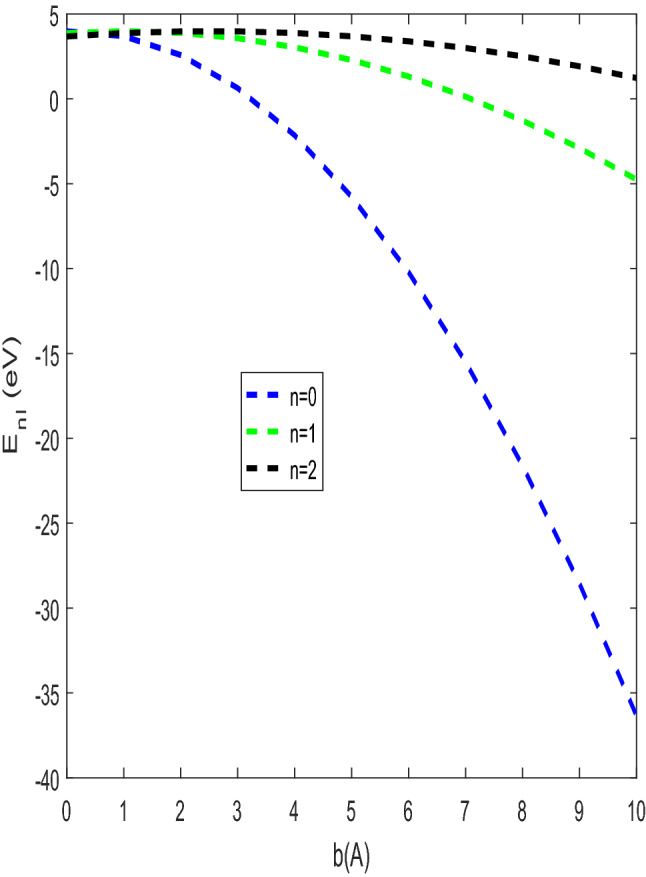
Energy $E_{n,\ell }$ against the $b$ with $\mu = \hbar = \ell = 1,$ $b_{h} = 0.001$ Å, $C_{h} = 0.95,$ $r_{e} = 0.5$ Å and $D_{e} = 10\;{\text{eV}}.$

The shape of the modified Tietz–Hua potential and the actual Tietz–Hua potential are shown in Fig. 1. The variation of energy eigenvalues against the optimization parameter is shown in Fig. 2. The energy eigenvalues varies directly with the optimization parameter for all quantum states. The energy at all states are bounded and tends to be equal at $C_{h} \ge 0.5.$ In Fig. 3, the behaviour of energy eigenvalues with $b_{h}$ was examined. The observed features in Fig. 2 were also seen in Fig. 3 except that at $b_{h} \ge 0.45,$ the energy were unbounded. In Fig. 4, the energy varies inversely with the modified parameter. As the modified parameter increases, the energy at the ground state and the first excited state were found to be bounded.

**Figure 5:**
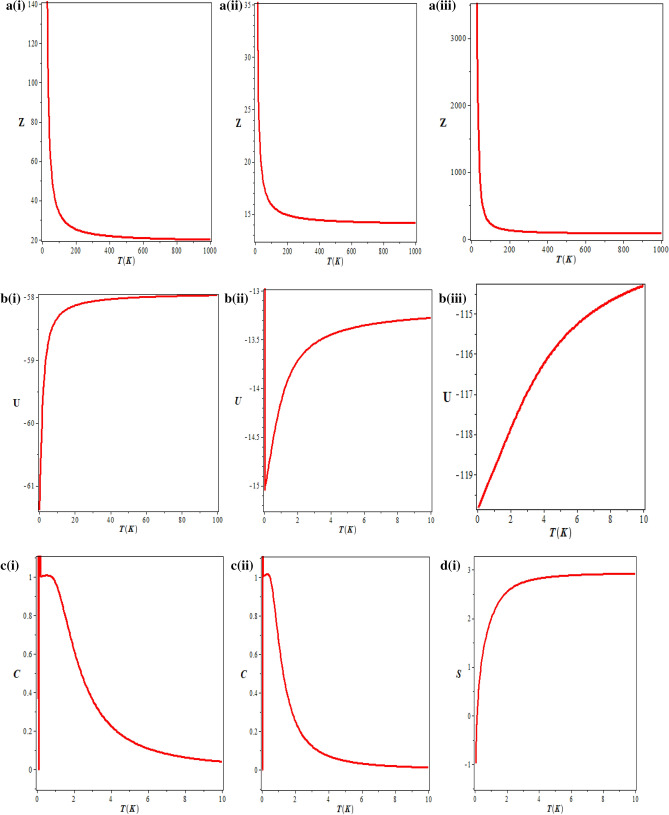

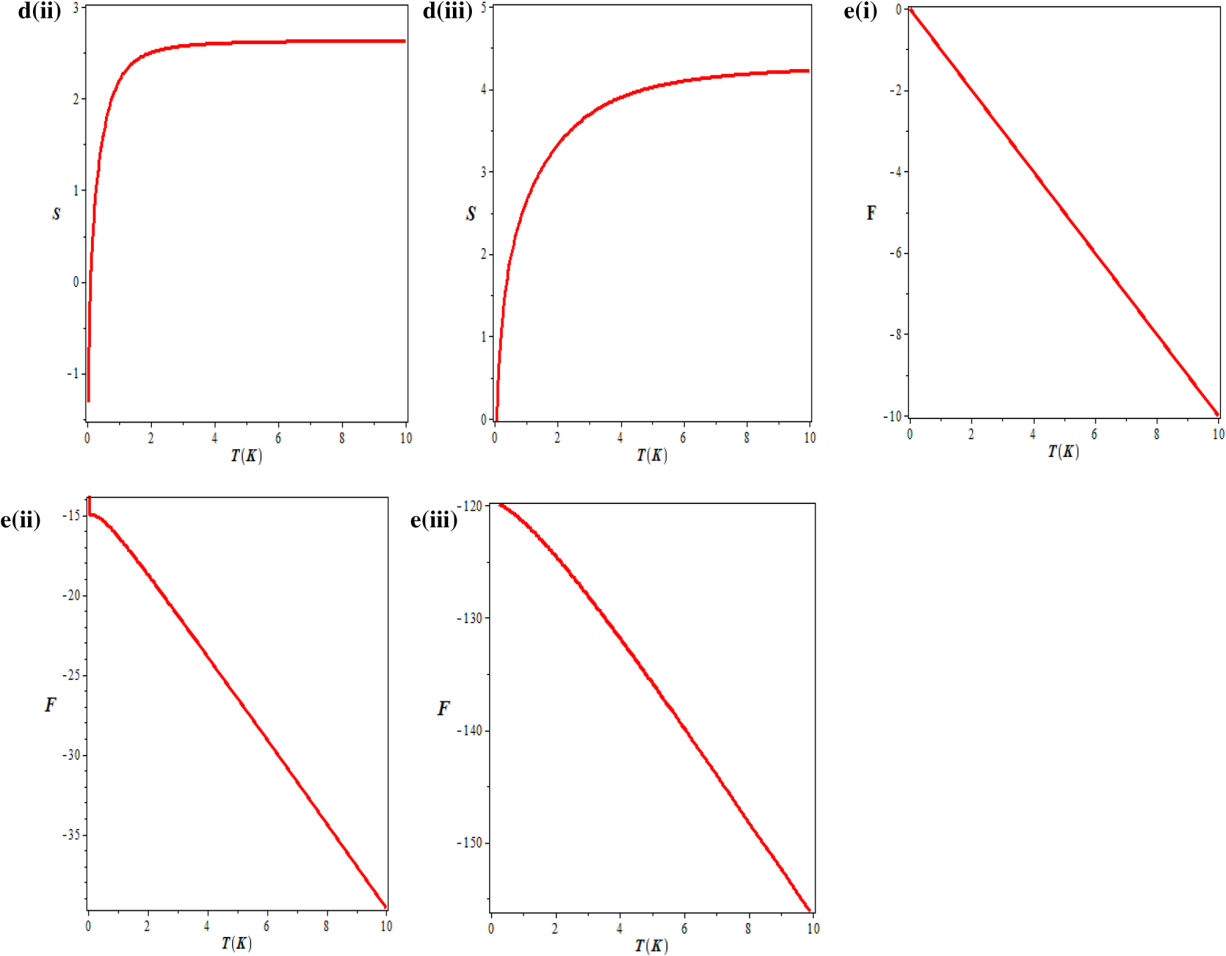
(**a**)(i) Vibrational partition function against temperature for HF. (**a**) (ii) Vibrational partition function against temperature for H_2_. (**a**) (iii) Vibrational partition function against temperature for CO. (**b**) (i) Vibrational mean energy against temperature for HF. (**b**) (ii) Vibrational mean energy against temperature for H_2_. (**b**) (iii) Vibrational mean energy against temperature for CO. (**c**) (i) Vibrational specific heat capacity against temperature for HF. (**c**) (ii) Vibrational specific heat capacity against temperature for H_2_. (**d**) (i) Vibrational entropy against temperature for HF. (**d**) (ii) Vibrational entropy against temperature for H_2_. (**d**) (iii) Vibrational entropy against temperature for CO. (**e**) (i) Vibrational free energy against temperature for HF. (**e**) (ii) Vibrational free energy against temperature for H_2_. (**e**) (iii) Vibrational free energy against temperature for CO.

We have also studied the thermodynamic properties of modified Tietz–Hua potential model, with a temperature dependent partition function being determined first. The thermodynamic properties such as “mean energy, specific heat capacity, free energy and entropy” were obtained from the calculated partition function. Figure 5a–e showed the variation of thermal properties against temperature for Hydrogen Fluoride (HF), Hydrogen molecule (H_2_) and Carbon (II) oxide (CO).

In Fig. 5a(i), Fig. 5a(ii) and Fig. 5a(iii) respectively, the variation of vibrational partition function against temperature for HF, H_2_ and CO are shown. It is noted that the vibrational partition function decrease exponentially with temperature at certain temperature range for the three molecules studied. At higher temperatures, the vibrational partition function remains constant. This behaviour is attributed to the three molecules. In Fig. 5b(i), Fig. 5b(ii) and Fig. 5b(iii), the behaviour of mean energy against the absolute temperature for HF, H_2_ and CO respectively are shown. Contrary to the behaviour of the partition function, the vibrational mean energy increases exponentially with the absolute temperature. The vibrational specific heat capacity is plotted against absolute temperature in Fig. 5c(i) and Fig. 5c(ii) for HF and H_2_ respectively. Contrary to the normal behaviour, the vibrational specific heat capacity decreases monotonically with increase in temperature for certain range of temperature. However, as the temperature gets higher, the specific heat capacity tends to be constant. This could be probably due to absorption of heat by the environment. The behaviour of the vibrational entropy with temperature is examined in Fig. 5d(i), Fig. 5d(ii) and Fig. 5d(iii) respectively for HF, H_2_ and CO. Within a temperature range of $0 \le T \le 2,$ there is a sharp increase in the entropy of the system for all the molecules. Outside this range, the vibrational entropy becomes constant. A critical observation from the Figures reals that CO with highest values in most of the spectroscopic parameters has the highest entropy at every value of the temperature. Figure 5e(i), Fig. 5e(ii) and Fig. 5e(iii) respectively, showed the variation of the vibration free energy with absolute temperature for HF, H_2_ and CO. The vibrational free energy decreases linearly with linear increase in absolute temperature. At absolute zero, the vibration free energy for HF is 0, H_2_ is − 15 K while CO is about − 117 K (156 °C).

The spectroscopic parameters for the model diatomic molecules used in this work were given in Table 1. In Table 2, the energy eigenvalues for four diatomic molecules studied were presented. The values were obtained by inserting the numerical values of the spectroscopic parameters in Table 1 into Eq. (31) and the programme was run with MATLAB 7.0. In Table 3, the comparison of experimental value with the calculated values for $Cs_{2} \left( {3^{3} \sum_{g}^{ + } } \right)$ molecule with $C_{h} = 0.01,$ $\omega_{e} = 28.8918\;{\text{cm}}^{ - 1} ,$ $r_{e} = 5.347420$Å and $D_{e} = 2722.28\;{\text{cm}}^{ - 1}$ from parametric Nikiforov-Uvarov method (ref.^39^) and the present calculation (supersymmetric approach) have been presented. The present results is in good agreement with the observed value. The numerical values for energy eigenvalues of the modified Tietz–Hua potential and the actual Tietz–Hua potential were presented in Table 4. For a very small value of the modified parameter, the energy eigenvalues of the modified Tietz–Hua potential is the same as the energy of the actual Tietz–Hua potential for at least five significant figures.

**Table 1:** Model parameters for diatomic molecules used in this work^13^.

Molecules	$C_{h}$	$\mu /10^{ - 23} (g)$		$b_{h} \left( {\dot{A}} \right)$		$r_{e} \left( {\dot{A}} \right)$	$D_{e} (cm^{ - 1} )$	
$O_{2} \left( {X^{3} \sum_{g}^{ + } } \right)$	0.027262	1.337		2.59103		1.207	42,041	
$NO\left( {X^{2} \Pi_{r} } \right)$	0.013727	1.249		2.71559		1.151	53,341	
$N_{2} \left( {X^{1} \sum_{g}^{ + } } \right)$	− 0.032325	1.171		2.78585		1.097	79,885	
$I_{2} \left( {X\left( {O_{g}^{2} } \right)} \right)$	− 0.139013	10.612		2.12343		2.666	12,547

**Table 2:** Bound-states energy eigenvalues of the molecules studied in this work for various states.

State	$b$	$O_{2} \left( {X^{3} \sum_{g}^{ + } } \right)$	$NO\left( {X^{2} \Pi_{r} } \right)$	$N_{2} \left( {X^{1} \sum_{g}^{ + } } \right)$	$I_{2} \left( {X\left( {O_{g}^{2} } \right)} \right)$
1s	0.05	5.079847490	6.144209789	− 9.960854035	− 1.187204187
	0.10	4.915358989	5.931830702	− 10.43369853	− 1.254403683
	0.15	4.749210578	5.717330873	− 10.90977300	− 1.321874438
2s	0.05	11.63173090	14.38422165	− 33.44603879	− 3.632904154
	0.10	11.56083000	14.28522802	− 34.05205250	− 3.707790719
	0.15	11.48830888	14.18413819	− 34.66122335	− 3.782937557
2p	0.05	5.770705550	7.176272301	− 9.446571039	− 1.185329504
	0.10	5.608205637	6.968337473	− 9.920964379	− 1.252582258
	0.15	5.444045814	6.758281901	− 10.39858771	− 1.320106283
3s	0.05	13.01931639	16.62871466	− 62.66223234	− 6.345187714
	0.10	13.03868668	16.64114875	− 63.39696081	− 6.427303661
	0.15	13.05647502	16.65151078	− 64.13477602	− 6.509669552
3p	0.05	12.21052211	15.17875174	− 32.99562289	− 3.632497076
	0.10	12.14156226	15.08415092	− 33.60315052	− 3.707434880
	0.15	12.07098219	14.98745390	− 34.21383530	− 3.782632967
3d	0.05	7.145274627	9.212456992	− 8.420233169	− 1.181611503
	0.10	6.986751891	9.013410680	− 8.897724208	− 1.248970788
	0.15	6.826569245	8.812243624	− 9.378445233	− 1.316601362

**Table 3:** Comparison of RKR data with calculated energies for $Cs_{2} \left( {3^{3} \sum_{g}^{ + } } \right)$ molecule.

$v$	RKR value ${\text{cm}}^{ - 1}$^38^	Present value ${\text{cm}}^{ - 1}$	Horchani et al ${\text{cm}}^{ - 1}$^39^
0	19,477.5507	19,477.5596	19,477.5587
1	19,506.2939	19,506.2924	19,506.2910
2	19,534.8916	19,534.8806	19,534.8758
3	19,563.347	19,563.3241	19,563.3072
4	19,591.6634	19,591.6199	19,591.5852
5	19,619.8441	19,619.8198	19,619.7097
6	19,647.8922	19,647.8392	19,647.6807
7	19,675.8110	19,675.6530	19,675.4983
8	19,703.6037	19,703.3007	19,703.1624
9	19,731.2736	19,730.9896	19,730.6731
10	19,758.8239	19,758.4021	19,758.0303
11	19,786.2579	19,785.7655	19,785.2341
12	19,813.5788	19,813.4431	19,812.2844

**Table 4:** Comparison of the energy eigenvalues (in eV) of the modified Tietz–Hua and actual Tietz–Hua potentials with $a = \hbar = \mu = 1,$ $r_{e} = 0.5$ Å,$b_{h} = 0.55$ Å, $b = 0.001$ Å, $C_{h} = 0.95$ and $D_{e} = 10eV.$

$v$	Modified Morse	Morse
0	9.050046340	9.051184909
1	9.949720443	9.949860955
2	9.969687015	9.969612361
3	9.774964556	9.774810138
4	9.461798638	9.461606144
5	9.055559076	9.055345496
6	8.565270611	8.565044146
7	7.994796585	7.994561676
8	7.346015067	7.345774325
9	6.619928221	6.619683283
10	5.817110437	5.816862380
11	4.937910111	4.937659672
12	3.982548517	3.982296219
13	2.951171668	2.950917889
14	1.843879061	1.843624085
15	0.660740395	0.660484438

## Conclusion

Using the supersymmetric approach, the energy equation and its corresponding wave function were obtained under the modified Tietz–Hua potential. The effect of the energy of modified Tietz–Hua potential is only different from that of the actual Tietz–Hua potential when the modified parameter is large. It is noted that the effect of temperature on the thermal properties differ. The results of $Cs_{2} \left( {3^{3} \sum_{g}^{ + } } \right)$ molecule in the present work (from supersymmetric approach) are in better agreement compared to the result obtained in ref.^39^ using parametric Nikiforov–Uvarov method.
